# Psychological Mediators of the Relationship Between Menopausal Symptoms and Health-Promoting Behaviors in Middle-Aged Women

**DOI:** 10.3390/healthcare14091210

**Published:** 2026-04-30

**Authors:** Jungmi Kang

**Affiliations:** Department of Nursing, Sun Moon University, Asan 31460, Republic of Korea; jung88922@naver.com

**Keywords:** menopausal symptoms, health-promoting behaviors, self-efficacy, self-acceptance, aging anxiety

## Abstract

**Background/Objectives:** Menopausal symptoms may negatively influence health-promoting behaviors in middle-aged women, but the psychological mechanisms underlying this association remain unclear. This study examined whether self-efficacy, self-acceptance, and aging anxiety mediate the relationship between menopausal symptoms and health-promoting behaviors. **Methods:** A cross-sectional survey was conducted among 114 middle-aged women. Data were analyzed using Pearson’s correlation analysis and a parallel multiple mediation model using Hayes’ PROCESS macro (Model 4) with bootstrapping (5000 resamples). **Results:** Menopausal symptoms were negatively correlated with self-efficacy, self-acceptance, and health-promoting behaviors and positively correlated with aging anxiety. Menopausal symptoms had a significant total effect on health-promoting behaviors (B = −0.126, *p* < 0.05), but the direct effect became non-significant after including the mediators (B = −0.006, 95% CI [−0.120, 0.111]). Significant indirect effects were observed through self-efficacy (B = −0.057, 95% CI [−0.121, −0.006]) and self-acceptance (B = −0.040, 95% CI [−0.074, −0.003]), whereas aging anxiety was not significant. The model explained 38.0% of the variance in health-promoting behaviors. **Conclusions:** The findings suggest that self-efficacy and self-acceptance play important mediating roles in the relationship between menopausal symptoms and health-promoting behaviors, highlighting the importance of psychological resources in the health management of middle-aged women.

## 1. Introduction

Women in midlife experience menopause, a transitional period characterized by simultaneous physical and emotional changes. Menopausal symptoms arise primarily from hormonal fluctuations and commonly include pain, weight gain, sleep disturbances, and mood changes [1]. These changes are not merely physiological events but can substantially influence daily functioning and health-related behaviors [2]. When menopausal symptoms are not adequately managed, their severity may increase, potentially leading to a decline in overall well-being and quality of life [3].

Health-promoting behaviors refer to activities performed by individuals to maintain and improve health, including regular physical activity, balanced nutrition, and effective stress management [4]. During the menopausal transition, engagement in health-promoting behaviors plays a critical role in alleviating symptoms and maintaining long-term health [5]. However, as menopausal symptoms become more severe, physical discomfort and emotional distress may increase, potentially hindering the adoption or maintenance of healthy behaviors [6]. Some studies have reported that severe menopausal symptoms are associated with decreased engagement in health-promoting behaviors [6], whereas other studies have suggested that heightened health awareness during this period may lead to increased health-related activities [7]. These inconsistent findings indicate that the relationship between menopausal symptoms and health-promoting behaviors may be complex and influenced by additional factors. Therefore, a more comprehensive understanding of this relationship is needed.

Psychological factors have been suggested as important explanatory variables in this context. Self-efficacy, defined as an individual’s belief in their ability to successfully perform a specific behavior, has been widely recognized as a key predictor of health behaviors [8,9]. Individuals with higher self-efficacy are more likely to persist in behaviors even when challenges arise, and this tendency has been associated with greater engagement in various health-promoting activities, including physical activity, dietary regulation, and stress management [9]. Among middle-aged women, higher levels of self-efficacy have been associated with more active coping with menopausal symptoms and increased participation in health management behaviors [10]. This relationship is consistent with Bandura’s social cognitive theory, which suggests that individuals who believe in their capability to perform a behavior are more likely to initiate and sustain health-related actions [8,11].

Self-acceptance is another important psychological resource in the process of adapting to menopause. Self-acceptance, identified by Ryff as a central component of psychological well-being [12], is associated with developing a positive attitude toward aging and bodily changes [13]. Such positive psychological resources not only facilitate the adoption of health-promoting behaviors but may also act as protective factors that buffer against reductions in health behaviors under stressful conditions [14,15].

Aging anxiety refers to the fear and concern individuals experience regarding the physical and social changes associated with aging [16]. Individuals with higher levels of aging anxiety may develop more negative perceptions of bodily changes, which may lead to avoidance or reduced engagement in health-promoting behaviors [17]. Although previous research has suggested that aging anxiety may be associated with health-promoting behaviors [17], research examining the mediating role of aging anxiety in the relationship between menopausal symptoms and health-promoting behaviors remains limited.

Previous studies have examined the relationships among menopausal symptoms, health-promoting behaviors, and psychological factors; however, most studies have focused on individual variables rather than considering these factors within an integrated framework. In particular, empirical evidence examining whether psychological factors mediate the relationship between menopausal symptoms and health-promoting behaviors remains limited. Furthermore, studies applying a parallel mediation model that simultaneously includes self-efficacy, self-acceptance, and aging anxiety are scarce.

This study is based on a socio-cognitive perspective that integrates cognitive (self-efficacy) and emotional (self-acceptance and aging anxiety) factors, providing a more comprehensive framework to understand the mechanisms underlying health-promoting behaviors.

Therefore, this study aimed to examine whether self-efficacy, self-acceptance, and aging anxiety mediate the relationship between menopausal symptoms and health-promoting behaviors among middle-aged women. By identifying the psychological mechanisms underlying this relationship, the present study sought to provide evidence that may inform the development of psychological intervention strategies to promote health-promoting behaviors and support effective health management during the menopausal transition.

## 2. Materials and Methods

### 2.1. Study Design

This study was conducted as a quantitative cross-sectional study based on secondary data analysis to examine the mediating roles of psychological factors, including self-efficacy, self-acceptance, and aging anxiety, in the relationship between menopausal symptoms and health-promoting behaviors. Previously collected data from an online survey were used to test the study hypotheses. This design was considered appropriate for examining the associations among variables and exploring potential mediating mechanisms within the study context.

### 2.2. Participants

The participants in this study were middle-aged women aged 40 to 59 years who voluntarily participated in an online survey and responded to questionnaires regarding menopausal symptoms, health-promoting behaviors, self-efficacy, self-acceptance, and aging anxiety.

Participants were not restricted to postmenopausal women, as the menopausal transition is a continuous process that includes premenopausal and perimenopausal stages. Women in these stages may also experience menopausal symptoms and related psychological changes. Therefore, individuals who had not yet reached menopause were included to capture a broader range of menopausal experiences.

The required sample size was calculated using the G*Power 3.1 program. Based on the statistical analysis method of this study, the minimum sample size was determined to be 102 participants with an effect size of 0.15, a significance level of 0.05, a statistical power of 0.90, and three predictor variables. Considering an estimated dropout rate of approximately 15%, a total of 118 participants were recruited for the online survey. After excluding four incomplete responses, data from 114 participants were included in the final analysis.

Data were collected through an online survey conducted over a 15-day period from 1 January to 15 January 2025.

### 2.3. Measures

To improve clarity and consistency, a summary of the measurement instruments used in this study is presented in Table 1.

#### 2.3.1. Menopausal Symptoms

Menopausal symptoms were measured using the Menopause Rating Scale (MRS) developed by Heinemann et al. [18]. The instrument consists of 11 items, each rated on a 5-point scale ranging from 0 (“no symptoms”) to 4 (“very severe”). Higher scores indicate more severe menopausal symptoms. The reliability of the instrument has been previously established. In the present study, internal consistency was confirmed with a Cronbach’s α of 0.90.

#### 2.3.2. Self-Efficacy

Self-efficacy was measured using the Self-Efficacy Scale originally developed by Sherer et al. [19] and later translated into Korean by Hong [20]. The instrument consists of 23 items, each rated on a 5-point Likert scale ranging from 1 (“strongly disagree”) to 5 (“strongly agree”). Total scores range from 23 to 115, with higher scores indicating greater self-efficacy. The original instrument reported a reliability of Cronbach’s α = 0.78, and the Korean version reported Cronbach’s α = 0.77. The reliability of the instrument has been previously established in both the original and Korean versions. In the present study, internal consistency was confirmed with a Cronbach’s α of 0.91.

#### 2.3.3. Self-Acceptance

Self-acceptance was measured using the Revised Unconditional Self-Acceptance Questionnaire developed by Chamberlain and Haaga [21] and later translated and validated in Korean by Chu and Lee [22]. The instrument consists of 15 items, each rated on a 5-point Likert scale ranging from 1 (“strongly disagree”) to 5 (“strongly agree”). Total scores range from 15 to 75, with higher scores indicating greater self-acceptance. The original instrument reported the reliability of Cronbach’s α = 0.72, and the Korean version reported Cronbach’s α = 0.77. The reliability of the instrument has been previously established. In the present study, internal consistency was confirmed with a Cronbach’s α of 0.81.

#### 2.3.4. Aging Anxiety

Aging anxiety was measured using the Aging Anxiety Scale developed by Lee and Yoo [23]. The instrument consists of 19 items, each rated on a 5-point Likert scale ranging from 1 (“strongly disagree”) to 5 (“strongly agree”). Total scores range from 19 to 95, with higher scores indicating greater aging anxiety. The original instrument reported a reliability of Cronbach’s α = 0.91. The reliability of the instrument has been previously established. In the present study, internal consistency was confirmed with a Cronbach’s α of 0.93.

#### 2.3.5. Health-Promoting Behaviors

Health-promoting behaviors were measured using the Health-Promoting Lifestyle Profile II (HPLP-II) developed by Walker et al. [24] and later translated into Korean by Seo and Ha [25]. The instrument consists of 50 items, each rated on a 4-point Likert scale ranging from 1 (“never”) to 4 (“always”). Higher scores indicate greater engagement in health-promoting behaviors. The original instrument reported the reliability of Cronbach’s α = 0.92, and the Korean version also reported Cronbach’s α = 0.92. The reliability of the instrument has been previously established. In the present study, internal consistency was confirmed with a Cronbach’s α of 0.95.

### 2.4. Statistical Analysis

Statistical analyses were performed using IBM SPSS Statistics version 26.0 (IBM Corp., Armonk, NY, USA). Descriptive statistics, including frequencies, percentages, means, and standard deviations, were used to describe the general characteristics of the participants and the study variables. Pearson’s correlation coefficients were calculated to examine the relationships among the major variables. To examine the mediating effects of self-efficacy, self-acceptance, and aging anxiety on the relationship between menopausal symptoms and health-promoting behaviors, Hayes’ PROCESS macro for SPSS (Model 4) was employed. This approach is based on ordinary least squares regression and bootstrapping procedures; therefore, conventional model fit indices (e.g., CFI, RMSEA) are not applicable in this analysis. The significance of the indirect effects was tested using bootstrapping with 5000 resamples. A mediating effect was considered significant when the 95% confidence interval (CI) of the indirect effect did not include zero. The level of statistical significance was set at *p* < 0.05.

### 2.5. Ethical Considerations

This study was conducted after obtaining exemption approval from the Institutional Review Board (IRB) of Sun Moon University (IRB No. SM-202511-028-1). Participants were informed about the purpose and procedures of the study, and informed consent was obtained prior to participation in the online survey. The collected data were used solely for research purposes, and all data were anonymized to ensure participants’ confidentiality and privacy.

## 3. Results

### 3.1. Participant Characteristics

The general characteristics of the 114 participants are presented in Table 2. Participants aged 40–49 years accounted for 66 (57.9%), while those aged 50–59 years accounted for 48 (42.1%). Regarding education level, 59 (51.8%) had a college degree or higher, and 55 (48.2%) had a high school education or lower. Most participants were married (n = 103, 90.4%). Seventy-five participants (65.8%) were employed, whereas 39 (34.2%) were unemployed. In terms of monthly household income, 64 participants (56.1%) reported an income of ≥4 million KRW, and 50 (43.9%) reported <4 million KRW. Most participants had at least one child (n = 107, 93.9%), while 7 (6.1%) had no children. Regarding menstrual status, 63 (55.3%) reported regular menstruation, 29 (25.4%) were menopausal, and 22 (19.3%) reported irregular menstruation.

### 3.2. Differences in Study Variables According to General Characteristics

Differences in study variables according to general characteristics are presented in Table 3.

### 3.3. Descriptive Statistics of Study Variables

The means and standard deviations of the major variables are presented in Table 4. The mean score for menopausal symptoms was 0.95 (0.69). Self-efficacy had a mean of 3.45 (0.53). Self-acceptance had a mean of 3.24 (0.45). Aging anxiety showed a mean of 2.99 (0.66). The mean score for health-promoting behaviors was 2.30 (0.41).

### 3.4. Correlations Among Study Variables

Pearson correlation analyses were conducted to examine the relationships among the major variables, as presented in Table 5. Menopausal symptoms were negatively correlated with health-promoting behaviors (r = −0.211, *p* = 0.024). Menopausal symptoms were positively correlated with aging anxiety (r = 0.377, *p* < 0.001) and negatively correlated with self-efficacy (r = −0.225, *p* = 0.016) and self-acceptance (r = −0.291, *p* = 0.002). Health-promoting behaviors were positively correlated with self-efficacy (r = 0.553, *p* < 0.001) and self-acceptance (r = 0.444, *p* < 0.001) and were negatively correlated with aging anxiety (r = −0.380, *p* < 0.001).

### 3.5. Parallel Mediation Analysis

A parallel multiple mediation analysis was conducted using the PROCESS macro (Model 4) with 5000 bootstrap resamples, and the results are presented in Table 6. Menopausal symptoms had a significant total effect on health-promoting behaviors (B = −0.126, 95% CI [−0.244, −0.008]). However, when self-efficacy, self-acceptance, and aging anxiety were included in the model, the direct effect of menopausal symptoms on health-promoting behaviors was not statistically significant (B = −0.006, 95% CI [−0.120, 0.111]).

The indirect effect through self-efficacy was significant (B = −0.057, 95% CI [−0.121, −0.006]), as was the indirect effect through self-acceptance (B = −0.040, 95% CI [−0.074, −0.003]). In contrast, the indirect effect through aging anxiety was not significant (B = −0.023, 95% CI [−0.067, 0.028]). These findings suggest that self-efficacy and self-acceptance mediated the relationship between menopausal symptoms and health-promoting behaviors, whereas aging anxiety did not function as a significant mediator. The significance of the indirect effects was determined based on whether the 95% bootstrap confidence intervals included zero.

**Figure 1:**
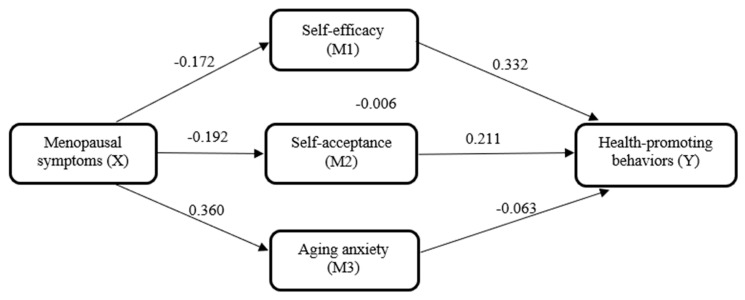
Parallel mediation model of the study variables. Values represent unstandardized regression coefficients (B) for each path; non-significant paths are indicated where applicable.

The overall model explained 38.0% of the variance in health-promoting behaviors (R^2^ = 0.380). The final path model is illustrated in Figure 1.

## 4. Discussion

This study examined the parallel mediating roles of psychological resources—self-efficacy, self-acceptance, and aging anxiety—in the relationship between menopausal symptoms and health-promoting behaviors among middle-aged women, based on a socio-cognitive framework that integrates cognitive and emotional factors influencing health behaviors. The findings indicated that menopausal symptoms were negatively associated with health-promoting behaviors; however, the direct effect became non-significant when the mediating variables were included in the model, whereas the indirect effects were significant, suggesting that the influence of menopausal symptoms on health-promoting behaviors may be indirectly explained through psychological mechanisms rather than reflecting a direct causal relationship. In particular, self-efficacy and self-acceptance were identified as significant mediators, whereas aging anxiety did not demonstrate a significant mediating pathway. These findings highlight the importance of psychological resources in understanding health-promoting behaviors among middle-aged women experiencing menopausal symptoms.

Self-efficacy played a significant mediating role in the relationship between menopausal symptoms and health-promoting behaviors. This finding suggests that menopausal symptoms may be associated with health-promoting behaviors indirectly by affecting individuals’ beliefs in their ability to manage their health. Self-efficacy has been widely recognized as a key psychological resource influencing behavioral choices and persistence [8] and has consistently been identified as an important predictor of health behavior change and maintenance in various health-related behaviors, including physical activity and sedentary behavior [26,27]. These results suggest that the physical and emotional changes experienced during menopause may influence middle-aged women’s perceived capability to engage in health management behaviors. These findings are consistent with previous studies indicating that individuals with higher self-efficacy are more likely to initiate and maintain health-promoting behaviors.

Similarly, self-acceptance was also identified as a significant mediator in the relationship between menopausal symptoms and health-promoting behaviors. This finding indicates that the physical changes and emotional discomfort associated with menopausal symptoms may influence individuals’ self-perception and level of self-acceptance, which may subsequently facilitate engagement in health-promoting behaviors. Self-acceptance refers to an attitude of positively recognizing and accepting one’s current state and experiences and is considered an emotional resource that promotes adaptive coping in stressful situations [28]. Previous studies have reported that self-acceptance is associated with mental health and subjective well-being and functions as a protective factor that enhances individuals’ adaptive functioning [29]. These findings suggest that interventions designed to help middle-aged women accept bodily changes and life transitions may contribute to improved health-promoting behaviors beyond approaches focusing solely on symptom reduction. This result suggests that emotional acceptance of menopausal changes may play an important role in supporting adaptive health behaviors during midlife.

In contrast, aging anxiety showed significant correlations with menopausal symptoms and health-promoting behaviors but did not demonstrate a significant mediating effect in the analytical model. This finding suggests that although aging anxiety may be related to health-related perceptions at the emotional level, it may have limited explanatory power in predicting actual health behavior performance. Previous research has reported that negative perceptions of aging are associated with health behavior participation; however, these relationships are often mediated or moderated by socio-cognitive factors [30]. Thus, cognitive resources such as perceived behavioral control or self-regulatory capacity may have a more proximal association with health behavior engagement than emotional attitudes toward aging, which may be influenced by broader social and cultural contexts. Taken together, these findings emphasize that active psychological resources, particularly self-efficacy and self-acceptance, play a more critical role in explaining health-promoting behaviors among middle-aged women experiencing menopausal symptoms.

Overall, the findings of this study suggest that psychological resources play a central role in linking menopausal symptoms to health-promoting behaviors among middle-aged women. These findings extend previous research by demonstrating that psychological mechanisms, particularly self-efficacy and self-acceptance, may explain how menopausal symptoms influence health-promoting behaviors. The integration of cognitive (self-efficacy) and emotional (self-acceptance) resources provides a more comprehensive understanding of the psychological processes underlying health behavior engagement during the menopausal transition.

These findings suggest that future health management strategies for middle-aged women may extend beyond focusing solely on alleviating menopausal symptoms and instead emphasize strengthening individuals’ acceptance of bodily changes and confidence in their ability to perform health-related behaviors. In addition, from a preventive perspective, broader socio-environmental factors such as education level, access to health information, and socioeconomic status may also play important roles in shaping health behaviors and may be considered in future intervention strategies. In particular, self-efficacy has been widely identified as a key cognitive factor influencing the initiation and maintenance of health behaviors [26,27], while self-acceptance has been suggested as an emotional resource that facilitates adaptive functioning in stressful situations [28,29]. Future research may explore integrating strategies to enhance self-efficacy and foster self-acceptance in the development of health promotion programs for middle-aged women.

This study has several limitations. First, because this study was based on a cross-sectional design using secondary data analysis, causal relationships among variables cannot be definitively established. Second, all variables were measured using self-report questionnaires, which may introduce potential response bias. In particular, the use of self-reported measures may raise concerns regarding common method bias, which should be considered when interpreting the findings. This may have influenced the observed associations among variables, potentially inflating or distorting the strength of the relationships. Third, the participants were limited to middle-aged women who participated in an online survey, which may limit the generalizability of the findings, particularly to middle-aged women with diverse socioeconomic, cultural, or clinical backgrounds. Additionally, the relatively small sample size may limit statistical power and the generalizability of the mediation effects. In addition, it should be noted that the analytical approach used in this study does not provide conventional model fit indices, as the mediation analysis was conducted using a regression-based PROCESS macro. Future research may employ longitudinal designs to better examine temporal relationships among menopausal symptoms, psychological resources, and health-promoting behaviors, thereby allowing for a clearer understanding of directionality and potential causal pathways. In addition, intervention studies may also be explored to develop and evaluate programs that integrate strategies to enhance self-efficacy and self-acceptance in order to promote health behaviors among middle-aged women.

## 5. Conclusions

This study examined the mediating roles of psychological factors in the relationship between menopausal symptoms and health-promoting behaviors among middle-aged women using a parallel mediation model based on secondary data analysis. The findings identified significant mediating roles of self-efficacy and self-acceptance in the relationship between menopausal symptoms and health-promoting behaviors. While aging anxiety was significantly associated with both menopausal symptoms and health-promoting behaviors, it did not demonstrate a significant mediating effect when psychological resources were considered.

These findings suggest that cognitive and emotional resources, particularly self-efficacy and self-acceptance, play a more important role than negative emotional responses in explaining engagement in health-promoting behaviors among middle-aged women. Health management strategies should therefore focus on strengthening individuals’ beliefs in their capacity to perform health behaviors and fostering adaptive acceptance of physical changes during midlife. Future health promotion interventions for middle-aged women may benefit from integrating strategies that enhance self-efficacy and foster self-acceptance to support sustainable health-promoting behaviors.

## Figures and Tables

**Table 1:** Summary of Measurement Instruments.

Variable	Instrument (Author, Year)	No. of Items	Scale	Description
Menopausal symptoms	MRS (Heinemann et al., 2004) [18]	11	0–4-point Likert scale	Assesses the severity of menopausal symptoms
Self-efficacy	Self-Efficacy Scale (Sherer et al., 1982; Hong, 1995) [19,20]	23	1–5-point Likert scale	Assesses perceived self-efficacy
Self-acceptance	Revised Unconditional Self-Acceptance Questionnaire (Chamberlain & Haaga, 2001; Chu & Lee, 2015) [21,22]	15	1–5-point Likert scale	Assesses unconditional self-acceptance
Aging anxiety	Aging Anxiety Scale (Lee & Yoo, 2013) [23]	19	1–5-point Likert scale	Assesses anxiety related to aging
Health-promoting behaviors	HPLP-II (Walker et al., 1987; Seo & Ha, 2001) [24,25]	50	1–4-point Likert scale	Assesses health-promoting lifestyle behaviors

**Table 2:** General characteristics of participants (N = 114).

Variable	Category	n (%)
Age	40–49	66 (57.9)
	50–59	48 (42.1)
Education	≤High school	55 (48.2)
	≥College	59 (51.8)
Marital status	Married	103 (90.4)
	Unmarried	4 (3.5)
	Other	7 (6.1)
Employment	Yes	75 (65.8)
	No	39 (34.2)
Monthly income	<4 million KRW	50 (43.9)
	≥4 million KRW	64 (56.1)
Number of children	≥1	107 (93.9)
	None	7 (6.1)
Menstrual status	Regular	63 (55.3)
	Irregular	22 (19.3)
	Menopause	29 (25.4)

**Table 3:** Differences in study variables by general characteristics (N = 114).

Variable	Category	Menopausal Symptoms		Self-Efficacy		Self-Acceptance		Aging Anxiety		Health-Promoting Behaviors	
		M (SD)	*p*	M (SD)	*p*	M (SD)	*p*	M (SD)	*p*	M (SD)	*p*
Age	40–49	0.82 (0.66)	0.017	3.45 (0.51)	0.891	3.24 (0.44)	0.964	2.92 (0.58)	0.167	2.26 (0.39)	0.293
	50–59	1.13 (0.69)		3.46 (0.55)		3.24 (0.48)		3.10 (0.74)		2.35 (0.43)	
Education	≤High school	0.91 (0.59)	0.574	3.35 (0.53)	0.045	3.09 (0.42)	0.001	3.08 (0.67)	0.184	2.18 (0.34)	0.003
	≥College	0.99 (0.77)		3.55 (0.51)		3.37 (0.44)		2.91 (0.64)		2.41 (0.44)	
Marital status	Married	0.96 (0.69)	0.647	3.43 (0.52)	0.268	3.23 (0.42)	0.540	2.98 (0.66)	0.571	2.29 (0.40)	0.811
	Unmarried	0.64 (0.52)		3.42 (0.53)		3.13 (0.40)		2.99 (0.49)		2.32 (0.30)	
	Other	0.99 (0.74)		3.76 (0.60)		3.41 (0.84)		3.25 (0.77)		2.39 (0.57)	
Employment	Yes	0.92 (0.61)	0.590	3.53 (0.52)	0.021	3.26 (0.46)	0.413	2.98 (0.66)	0.795	2.31 (0.41)	0.669
	No	1.00 (0.82)		3.29 (0.51)		3.19 (0.45)		3.01 (0.65)		2.28 (0.42)	
Monthly income	<4 million KRW	0.90 (0.65)	0.447	3.53 (0.53)	0.136	3.23 (0.53)	0.801	3.01 (0.70)	0.801	2.26 (0.38)	0.420
	≥4 million KRW	0.99 (0.71)		3.39 (0.51)		3.24 (0.39)		2.98 (0.62)		2.33 (0.43)	
Number of children	≥1	0.97 (0.70)	0.059	3.46 (0.53)	0.496	3.24 (0.46)	0.526	2.96 (0.64)	0.140	2.31 (0.41)	0.227
	None	0.64 (0.37)		3.32 (0.51)		3.15 (0.34)		3.44 (0.73)		2.15 (0.30)	
Menstrual status	Regular	0.86 (0.69)	0.312	3.45 (0.53)	0.865	3.26 (0.47)	0.716	2.92 (0.62)	0.072	2.31 (0.43)	0.277
	Irregular	1.05 (0.69)		3.50 (0.55)		3.17 (0.42)		3.28 (0.75)		2.18 (0.36)	
	Menopause	1.07 (0.67)		3.42 (0.50)		3.25 (0.44)		2.93 (0.62)		2.36 (0.39)	

Note. Values are presented as mean (standard deviation); all *p*-values are two-tailed.

**Table 4:** Descriptive statistics of study variables (N = 114).

Variable	N	Mean	SD	Min	Max	95% CI (Lower–Upper)
Menopausal symptoms	114	0.95	0.69	0.00	2.73	0.82–1.08
Self-efficacy	114	3.45	0.53	2.22	4.91	3.35–3.55
Self-acceptance	114	3.24	0.45	2.40	5.00	3.16–3.32
Aging anxiety	114	2.99	0.66	1.53	5.00	2.81–3.05
Health-promoting behaviors	114	2.30	0.41	1.60	3.46	2.23–2.38

Note. Values are presented as mean (standard deviation); CI = confidence interval.

**Table 5:** Correlations among study variables (N = 114).

Variable	Menopausal Symptoms r (*p*)	Self-Efficacy r (*p*)	Self-Acceptance r (*p*)	Aging Anxiety r (*p*)	Health-Promoting Behaviors r (*p*)
Menopausal symptoms	1				
Self-efficacy	−0.225 (*p* = 0.016)	1			
Self-acceptance	−0.291 (*p* = 0.002)	0.368 (*p* < 0.001)	1		
Aging anxiety	0.377 (*p* < 0.001)	−0.371 (*p* < 0.001)	−0.500 (*p* < 0.001)	1	
Health-promoting behaviors	−0.211 (*p* = 0.024)	0.553 (*p* < 0.001)	0.444 (*p* < 0.001)	−0.380 (*p* < 0.001)	1

**Table 6:** Total, direct, and indirect effects of menopausal symptoms on health-promoting behaviors (bootstrapping with 5000 resamples).

Effect/Path	B	SE	95% CI
Total effect (Menopausal symptoms → Health-promoting behaviors)	−0.1259	0.0550	[−0.2438, −0.0081]
Direct effect (Menopausal symptoms → Health-promoting behaviors)	−0.0055	0.0490	[−0.1203, 0.1106]
Total indirect effect	−0.1204	0.0377	—
Indirect effect via self-efficacy	−0.0572	0.0287	[−0.1213, −0.0065]
Indirect effect via self-acceptance	−0.0404	0.0185	[−0.0741, −0.0030]
Indirect effect via aging anxiety	−0.0228	0.0238	[−0.0671, 0.0280]

Note. CI = confidence interval. Indirect effects were estimated using bootstrapping with 5000 resamples.

## Data Availability

The data that support the findings of this study are available from the corresponding author upon reasonable request. The data are not publicly available due to privacy and ethical restrictions related to the protection of participants’ personal information.
